# Biochemical Risk Factors Associated With Hyperkalemia in Cirrhotic Patients

**DOI:** 10.7759/cureus.18356

**Published:** 2021-09-28

**Authors:** Versha Gurnani, Nomesh Kumar, Shayan Iqbal Khan, Muhammad Umair Nawaz, Hassan Ahmed, Sidra Naz, Aresha Masood Shah, Maha Jahangir

**Affiliations:** 1 Internal Medicine, Liaquat University of Medical and Health Sciences, Jamshoro, PAK; 2 Internal Medicine, Jinnah Sindh Medical University, Karachi, PAK; 3 Internal Medicine, Dow University of Health Sciences, Karachi, PAK; 4 Internal Medicine, University of Health Sciences, Lahore, PAK

**Keywords:** hyperkalemia, chronic liver disease, cirrhosis, cld, hepatitis

## Abstract

Introduction: Patients with cirrhosis suffer from fluid and electrolyte imbalance. The usually reported electrolyte disorders include hyperkalemia, hyponatremia, and hypokalemia. The regional data about the prevalence and risk factors associated with hyperkalemia in cirrhotic patients are not sufficient enough. The purpose of this study is to determine various risk factors associated with hyperkalemia, which will assist in the early detection of cirrhotic patients at risk of hyperkalemia.

Methods: This cross-sectional study was conducted in the internal medicine and gastroenterology departments of a tertiary care hospital in Pakistan from March 2021 to June 2021. Sonographically documented liver cirrhosis patients (n=500), of either gender and between the ages of 18 and 70 years, were enrolled in the study. After enrollment, patients’ demographics were noted in a self-structured questionnaire. Participant’s Child-Pugh score was also noted in the questionnaire. After a detailed history, 5 mL of venous blood was drawn in two vials via phlebotomy and send to the laboratory to measure serum potassium, creatinine, albumin, and bilirubin levels.

Result: Out of the total 500 participants, 101 (20.2%) participants had hyperkalemia. It was significantly more prevalent in participants with Child-Pugh C class and in those with a serum creatinine of more than 1.3 mg/dL. Similarly, it was more prevalent in participants with albumin levels less than 2.5 mg/dL.

Conclusion: Hyperkalemia is associated with Child-Pugh class C. It has a direct relationship with serum creatinine levels which is an indicator of renal function, and an inverse relationship with serum albumin levels, an indicator of hepatic synthetic function.

## Introduction

Chronic liver diseases (CLDs) eventually result in liver cirrhosis, causing normal liver architecture to convert into structurally abnormal nodules and characteristic tissue fibrosis [1]. Viral hepatitis, alcoholic liver disease, and nonalcoholic steatohepatitis are the most common causes of liver cirrhosis [2]. In addition to decreased life expectancy, several complications are faced by cirrhotic patients [3]. Varices, ascites, hepatic encephalopathy, hepatopulmonary hypertension, hepatocellular carcinoma, hepatorenal syndrome, spontaneous bacterial peritonitis, and coagulation disorders are the major complications of cirrhosis [4].

Furthermore, patients with CLD suffer from fluid and electrolyte imbalance. Usually reported electrolyte imbalances are hyperkalemia, hyponatremia, hypokalemia, respiratory alkalosis, and metabolic acidosis along with an excess accumulation of body fluids resulting in edema and ascites [5,6]. Deranged homeostasis of potassium, i.e., hyperkalemia, is often seen in patients with advanced cirrhosis. It is prevalent in 12-14% of cirrhotic patients, whereas the prevalence in the rest of the population is 2.1-7.0% [7-9].

Various etiologies can lead to hyperkalemia in cirrhotic patients. One of the causes is the use of potassium-sparing diuretics, such as spironolactone and eplerenone. The most important contributor to hyperkalemia in cirrhotic patients is decreased renal excretion, which explains its frequent association with increasing creatinine and blood urea nitrogen levels in azotemic patients [10,11]. Hyperkalemia can also be exacerbated by alcohol-induced rhabdomyolysis, gastrointestinal bleeding, or hemolysis in cirrhotic patients [12]. The purpose of this study is to determine various risk factors associated with hyperkalemia in cirrhotic patients. It will assist in the early detection of hyperkalemia in cirrhotic patients.

## Materials and methods

This cross-sectional study was conducted in the internal medicine and gastroenterology departments of a tertiary care hospital in Pakistan from March 2021 to June 2021. We enrolled 500 patients with confirmed diagnoses of liver cirrhosis made via sonography, of either gender and between the ages of 18 and 70 years. Patients on angiotensin-converting enzyme inhibitors and patients with chronic kidney disease were excluded from the study. Participants who had edema and needed spironalactone were also excluded from the study. Participants with a glomerular filtration rate of less than 60 mL/min/1.73 m^2^ for more than three months were labeled as chronic kidney disease patients.

Patients were enrolled via consecutive convenient non-probability sampling. Informed consent was taken before the enrollment of participants and they were informed about their right to withdrawal from the study at any given moment. Ethical review board approval was taken from the institute before the start of the study.

After enrollment, patients’ demographics were noted in a self-structured questionnaire. Participant’s Child-Pugh score was also noted in the questionnaire. After a detailed history, 5 mL of venous blood was drawn in two vials via phlebotomy and send to the laboratory to measure serum potassium, creatinine, albumin, and bilirubin levels. Participants were classified into two groups based on their serum potassium levels, i.e., hyperkalemia and normokalemia. Hyperkalemia was defined as a serum concentration of greater than 5.0 mmol/L [13].

Statistical analysis was done using the Statistical Packages for Social Sciences (SPSS), version. 23.0 (IBM Corporation, Armonk, New York, United States). Categorical data such as hyperkalemic and normokalemic participants were presented as frequency and percentage. Numerical data such as age and duration of cirrhosis were presented as mean and standard deviation. Unpaired T-test and chi-square were applied to compare the two groups (participants with hyperkalemia and normokalemia). A p-value of less than 0.05 meant that the difference between the groups is significant and the null hypothesis is void.

## Results

Out of the total 500 participants, 101 (20.2%) participants had hyperkalemia. The mean age and gender were comparable between the two groups. Time since diagnosis of cirrhosis was significantly more in the hyperkalemic compared to the normokalemic participants (13 ± 4 months vs. 12 ± 4 months; p-value: 0.02; Table 1).

**Table 1:** Demographics of participants mmol/L: milimoles per liter, SD: standard deviation

Demographics	Hyperkalemia (n=101)	Normokalemia (n=399)	p-value
Age in years (mean ± SD)	51 ± 9	52 ± 8	0.27
Gender			
Male	62 (61.3%)	239 (59.8%)	0.07
Female	39 (38.7%)	160 (40.2%)	
Duration since diagnosis of cirrhosis (in months)	13 ± 4	12 ± 4	0.02
Mean serum potassium level (mmol/L)	5.9 ± 0.9	4.1 ± 0.4	<0.0001

Hyperkalemia was significantly more prevalent in participants with class C Child-Pugh compared to class B and class A (72.2% vs. 15.8% vs. 11.8%; p-value: <0.00001). Participants with serum creatinine more than 1.3 mg/dL had more participants with hyperkalemia compared to participants with serum creatinine less than 1.3 mg/dL (88.1% vs. 11.9%; p-value: <0.0001). Hyperkalemia was also more prevalent in participants with serum albumin less than 2.5 mg/dL (65.3% vs. 34.6%; p-value: <0.0001) Similarly, hyperkalemia was more prevalent in participants with serum bilirubin more than 2.8 mg/dL (68.3% vs. 31.7%; p-value: 0.001; Table 2).

**Table 2:** Comparison of biochemical parameters of hyperkalemic and normokalemic participants mg/dL: milligrams per deciliter.

Clinical and biochemical features	Hyperkalemia (n=101)	Normokalemia (n=399)	p-value
Child-Pugh class			
A	12 (11.8%)	217 (54.3%)	<0.00001
B	16 (15.8%)	131 (32.8%)	
C	73 (72.2%)	51 (12.7%)	
Serum creatinine			
>1.3 mg/dL	89 (88.1%)	52 (13.0%)	<0.0001
<1.3 mg/dL	12 (11.9%)	347 (87.0%)	
Serum bilirubin			
>2.8 mg/dL	69 (68.3%)	201 (50.3%)	0.001
<2.8 mg/dL	32 (31.7%)	198 (49.7%)	
Serum albumin			
>2.5 mg/dL	35 (34.6%)	274 (68.6%)	<0.0001
<2.5 mg/dL	66 (65.3%)	125 (31.4%)	

## Discussion

Our study demonstrated that hyperkalemia is more prevalent in participants with Child-Pugh class C liver cirrhosis. It showed a direct relation with serum creatinine and bilirubin levels and an inverse relation with serum albumin levels. Prior studies have shown a similar pattern. Kidneys play a major role in the balance of electrolytes. Cai et al. found a direct correlation between potassium levels and serum creatinine (p-value: <0.001) and serum BUN (p-value: <0.001), whereas a negative correlation with serum sodium (p-value: <0.001); all of which are known contributors of renal dysfunction [6]. Another study by Maiwall et al. concluded that potassium imbalances are commonly seen in cirrhotic patients, and hyperkalemia is associated with increased serum creatinine and urea levels [7]. Mohsin et al. also stated that patients with CLD had a higher incidence of hyperkalemia as compared to their healthy counterparts [14]. Multivariate analysis showed that hyperkalemia was associated with high dose spironolactone, low albumin levels (p-value:0.017), and advanced cirrhosis with a high Child-Pugh score (p-value: 0.003) [15].

Diuretics such as spironolactone and eplerenone are commonly used in the management of cirrhotic patients and may lead to hyperkalemia [6]. Radó et al. reported that patients receiving conventional diuretics presented with hyperkalemia in 3.3% of testings instead of 48% in patients receiving spironolactone [10]. Furthermore, the study added that hyperkalemia was more severe in azotemic patients. Another contributor for hyperkalemia in cirrhotic patients is the increased outflow of potassium ions from the intracellular matrix to the extracellular fluid due to metabolic acidosis, hyperglycemia, and the use of beta-blockers [11]. According to an experiment conducted by Decaux et al., patients with compensated cirrhosis, three hours after the intake of an oral load of potassium, cirrhotic patients had higher potassium levels as compared to the healthy controls despite identical renal excretion. Along with hyperkalemia, an increase in the C-peptide levels was also seen, indicating a decrease in hepatic cellular uptake of potassium despite insulin hypersecretion [16].

Hyperkalemia is associated with poor prognosis in patients with cirrhosis as it is an indicator of worsening renal function [17]. According to Mezzano et al., hyperkalemia is an important sign of mortality in patients with acute on chronic liver failure (ACLF) and has shown an increased 90, 180, and 360-day mortality risk in these patients [18]. Cai et al. also reported that patients with ACLF had a higher 90-day mortality rate when acute kidney injury (AKI) was detected with hyperkalemia as compared to AKI without hyperkalemia [6]. In light of the significance of potassium levels in patients with cirrhosis, it is important to monitor them, timely identify derangements and provide treatment.

In asymptomatic patients with hyperkalemia, management is based on discontinuing potassium-sparing diuretics, decreasing the oral intake of potassium, avoiding massive transfusions, and correcting underlying acid-base disturbances [11]. In patients with symptomatic or severe hyperkalemia, treatment consists of intravenous (IV) calcium gluconate to stabilize cardiac membranes and IV glucose along with insulin to promote cellular uptake of potassium ions. Aerosolized beta-agonists can also be used to promote intracellular movement. To deplete the body of potassium, loop/thiazide diuretics and cation exchanging resins (kayexalate) can be administered. For refractory hyperkalemia, the last resort is hemodialysis [19].

This study has the following limitations. First, since the study was conducted in a single institute, the sample size was less diverse and limited. Second, since it was a cross-sectional study, the impact of hyperkalemia on the long-term prognosis of cirrhosis could not be assessed. Third, only biochemical parameters were assessed and other parameters such as age, weight, and medications were not assessed. Fourth, we did not differentiate between hyperkalemia caused by a higher creatinine level (>1.3 mg/dl) and hyperkalemia due to cirrhosis in the study participant, which may have impacted study results.

## Conclusions

Hyperkalemia is associated with severe liver cirrhosis. It has a direct relationship with serum creatinine levels, an indicator of renal function, and an inverse relation with serum albumin levels, an indicator of hepatic synthetic function. Early identification and correction of the underlying cause is required to decrease the mortality rates.
